# Mental health nurses’ attitudes, experience, and knowledge regarding routine physical healthcare: systematic, integrative review of studies involving 7,549 nurses working in mental health settings

**DOI:** 10.1186/s12912-019-0339-x

**Published:** 2019-04-26

**Authors:** Geoffrey L. Dickens, Robin Ion, Cheryl Waters, Evan Atlantis, Bronwyn Everett

**Affiliations:** 1 Professor Mental Health Nursing, Centre for Applied Nursing Research (CANR), Western Sydney University, Sydney, Australia; 2 South West Sydney Local Health District, Sydney, Australia; 3 Division of Mental Health Nursing and Counselling, Abertay University, Dundee, Scotland

**Keywords:** Mental health nurses, Emergency medicine, Deteriorating patient, Educational interventions, Attitudes, Knowledge

## Abstract

**Background:**

There has been a recent growth in research addressing mental health nurses’ routine physical healthcare knowledge and attitudes. We aimed to systematically review the empirical evidence about i) mental health nurses’ knowledge, attitudes, and experiences of physical healthcare for mental health patients, and ii) the effectiveness of any interventions to improve these aspects of their work.

**Methods:**

Systematic review in accordance with the Preferred Reporting Items for Systematic Reviews and Meta-Analyses guidelines. Multiple electronic databases were searched using comprehensive terms. Inclusion criteria: English language papers recounting empirical studies about: i) mental health nurses’ routine physical healthcare-related knowledge, skills, experience, attitudes, or training needs; and ii) the effectiveness of interventions to improve any outcome related to mental health nurses’ delivery of routine physical health care for mental health patients. Effect sizes from intervention studies were extracted or calculated where there was sufficient information. An integrative, narrative synthesis of study findings was conducted.

**Results:**

Fifty-one papers covering studies from 41 unique samples including 7549 mental health nurses in 14 countries met inclusion criteria. Forty-two (82.4%) papers were published since 2010. Eleven were intervention studies; 40 were cross-sectional. Observational and qualitative studies were generally of good quality and establish a baseline picture of the issue. Intervention studies were prone to bias due to lack of randomisation and control groups but produced some large effect sizes for targeted education innovations. Comparisons of international data from studies using the Physical Health Attitudes Scale for Mental Health Nursing revealed differences across the world which may have implications for different models of student nurse preparation.

**Conclusions:**

Mental health nurses’ ability and increasing enthusiasm for routine physical healthcare has been highlighted in recent years. Contemporary literature provides a base for future research which must now concentrate on determining the effectiveness of nurse preparation for providing physical health care for people with mental disorder, determining the appropriate content for such preparation, and evaluating the effectiveness both in terms of nurse and patient- related outcomes. At the same time, developments are needed which are congruent with the needs and wants of patients.

**Electronic supplementary material:**

The online version of this article (10.1186/s12912-019-0339-x) contains supplementary material, which is available to authorized users.

## Background

People with a mental disorder diagnosis are at more than double the risk of all-cause mortality than the general population. Most at risk are those with psychosis, mood disorder and anxiety diagnoses. Median length of life lost by this group is 10.1 years greater for people with a diagnosis of mental disorder than for general population controls, but mortality rates are significantly higher in studies which include inpatients [1]. While risk of unnatural causes of death, notably suicide, are greatly increased in this group, it is death from natural causes that remains responsible for the vast majority of mortality. In people with schizophrenia, for example, cardiovascular disease accounts for about one third of all deaths and cancer for one in six, while other common causes are diabetes mellitus, COPD, influenza, and pneumonia [2]. A relatively high rate of tobacco smoking in this group is implicated in significant increased mortality [3], as is obesity [4], exposure to high levels of antipsychotic pharmacological treatment [5], and mental disorder itself [1].

Accordingly, the physical health of patients with mental disorder has been prioritised, becoming the focus of guidelines for practitioners in general [6] and for mental health nurses and other clinical professionals specifically [7–9]. However, while policies and guidelines are necessary prerequisites of change they must also be implemented in practice if they are to have a positive effect; one of the key barriers to change implementation for mental health nurses has been identified as lack of confidence, skills, and knowledge [10]. Robson and Haddad ([11]: p.74) identified that surprisingly ‘modest attention’ had been paid to the issue of such attitudes and knowledge among nurses related to their role in physical health care provision, and developed the Physical Health Assessment Scale for mental health nurses (PHASe) in order to further investigate the phenomenon. Since then, there has been a tangible and growing response among mental health nursing academics and practitioners. In recent years, published literature reviews have covered a decade of UK-only research on the role of mental health nurses in physical health care [12], patients’ and professionals’ perceptions of barriers to physical health care for people with serious mental illness [13], the focus and content of nurse-provided physical healthcare for mental health patients [14], and the physical health of people with severe mental illness [15]. There has also been an upsurge in the amount of related empirical research. However, to date, no one has systematically reviewed this growing literature about mental health nurses’ attitudes towards, or their related knowledge and experience about providing routine physical healthcare. Further, studies about the effectiveness of interventions designed to improve their delivery of or attitudes to routine physical healthcare have not been systematically appraised. This is surprising given the known links between nurses’ attitudes and their implementation of evidence-based practice [16–18] and the centrality of measuring nurses’ attitudes to physical health care delivery in recent mental health nursing research on the topic [11, 19, 20].

In this context we have conducted a systematic review to identify, appraise, and synthesise existing evidence from empirical research literature about i) mental health nurses’ experience of providing physical healthcare for patients and about their related knowledge, skills, educational preparation, and attitudes; ii) the effectiveness of any interventions aimed at improving or changing mental health nurse-related outcomes; and iii) to identify implications for the future provision of relevant training and education, for policy, research, and practice. The specific review question being addressed therefore is: what is known from the international, English language, empirical literature about mental health nurses’ skills, knowledge, attitudes, and experiences regarding provision of physical healthcare.

## Methods

### Design

A systematic review of the literature following the relevant points of the Preferred Reporting Items for Systematic Reviews and Meta-Analyses [21].

### Search strategy

Since the review scope encompassed questions about experience *and* effectiveness a dual literature search strategy was developed. For studies about mental health nurses’ experience of delivering physical healthcare a Population Exposure Outcome (PEO) format review question was developed (Population: mental health nurses; Exposure: physical healthcare provision for patients or related training; Outcomes: experiential, social, educational, knowledge, or attitudinal terms, see Additional file 1: Table S1). For studies of the effectiveness of interventions to improve or change mental health nurse-related outcomes a Population Intervention Comparator Outcome (PICO) structure was implemented (Population: mental health nurses; Intervention: any intervention including physical health-related education, policy or guideline change; Comparator: any or none; Outcome: any) [22]. We searched five electronic databases: i) CINAHL, ii) PubMed, iii) MedLine, iv) Scopus, and v) ProQuest Dissertations and Theses using text words and MeSH terms. The references list of all included studies, together with those of relevant literature reviews, and the tables of contents of selected mental health nursing journals were hand searched. The search terms were informed by previous literature reviews on the subject of physical healthcare in mental health. The initial search was conducted in April 2018 and re-run in September 2018.

## Inclusion and exclusion criteria

Inclusion criteria for studies were English language accounts of empirical research which investigated mental health nurses’ experience of providing physical health care *or* examined the effectiveness of any intervention that aimed to improve outcomes related to the *provision* of physical healthcare. Thus, studies of interventions aimed at changing nursing practice, behaviour, knowledge, attitudes, or experiences were eligible, but not those which solely attempted to determine the effect of an intervention on nurses in terms of patient outcomes. While improvement in patient care and outcomes is clearly the desirable endpoint of any intervention on nurses, previous reviews have indicated that no good quality studies exist [23]. Additionally, studies were only eligible for inclusion where the practitioners involved comprised or included mental health or psychiatric nurses or mental health nursing students, or registered nurses whose practice was within mental health services. Included studies could have used any design or methodological approach. As in previous reviews, studies solely about mental health nurses providing care for people with alcohol/ drug misuse, or mental disorder/substance misuse dual diagnosis were not eligible. Studies about mental health nurses and the provision of emergency physical care or of their experience of providing care for the seriously deteriorating physical health of a patient were omitted as this is the subject of a separate review (Dickens et al. submitted).

### Data extraction

Information about the study title, author, publication year, data collection years, location (country), research objectives, aims or hypotheses, design, population, sample details and size, data sources, study variables (i.e. details of intervention) or other exposure, unit of analysis, and study findings were extracted from full text papers. Corresponding authors of included studies were contacted regarding any issues where clarification or additional data could aid the review.

Studies were categorised as interventional or observational. Intervention studies investigated the impact of an educational, policy, or practice intervention in terms of any mental health nurse- or nursing- related outcome, e.g., knowledge, attitudes, behaviour. Intervention studies were further sub-classified as *simulation studies* (as defined by Bland et al. ([24]: p.668) “a dynamic process involving the creation of a hypothetical opportunity that incorporates an authentic representation of reality, facilitates active student engagement and integrates the complexities of practical and theoretical learning with opportunity for repetition, feedback, evaluation and reflection”), traditional educational interventions (e.g., lectures, workshops, workbooks), or policy-level interventions (e.g., requiring nurses to follow some new policy or implement some new practice). Observational studies either described mental health nurse- or nursing- related outcomes and/or utilised case control designs to compare them with those of other occupational or professional groups and/or used qualitative methods.

### Study quality appraisal

The likelihood of bias in intervention studies was assessed against criteria described by Thomas et al. [25] and encompassed assessment of the likelihood of selection bias in the obtained sample, study design, potential confounders, blinding, potential for bias in data collection from invalid instrumentation, and participant retention (see Additional file 2: Table S2). Relevant items from the US Department of Health & Human Sciences NIH Quality Assessment Tool for Observational Cohort and Cross-Sectional Studies [26] were used to assess cross-sectional observational studies (see Additional file 3: Table S3). Qualitative descriptive studies were assessed using the Critical Appraisal Skills Programme [27] tool (See Additional file 4: Table S4). Multiple papers arising from single studies were quality assessed as a single entity. Study quality was initially undertaken independently by at least two of the team. A good level of inter-rater agreement was achieved (Cohen’s Kappa = 0.742 between pairs of raters). Disputed items were discussed by GD and CW and consensus achieved.

### Study synthesis

The available total and subscale data from those studies that conducted data collection via the Physical Healthcare Attitude Scale for mental health nurses (PHASe [11]), the only scale used across more than two studies, was tabulated and compared across studies using unpaired t-tests in QuickCalcs GraphPad software. Where individual item mean and dispersion scores were unavailable estimates were calculated as follows: the mean mean (i.e., Σ means / *n* means) and the estimated standard deviation (the square root of the average of the variances [28]). Also, and where available, dichotomised data (‘Strongly agree’ or ‘agree’ responses versus all other responses) from the multiple studies using the 14-item PHASe scale investigating self-reported current involvement in aspects of physical healthcare was tabulated and subjected to Chi-squared analysis. Significant cross-study differences of means and proportions involved all subscale or item data for each study being compared with the corresponding subscale or item from the original study development sample, ‘the reference group’ [11].

Where available, effect sizes for correlational, interventional, or difference-related outcomes from studies were extracted or, where sufficient information presented, calculated. Where sufficient information was not presented we attempted to contact the corresponding author for clarification. Appropriate effect size statistics were calculated using an online resource [29]. All other information from study results was subject to a qualitative synthesis conducted by author 1 and subsequently refined and agreed by all of the authors.

## Results

### Study settings and participants

**Fig. 1:**
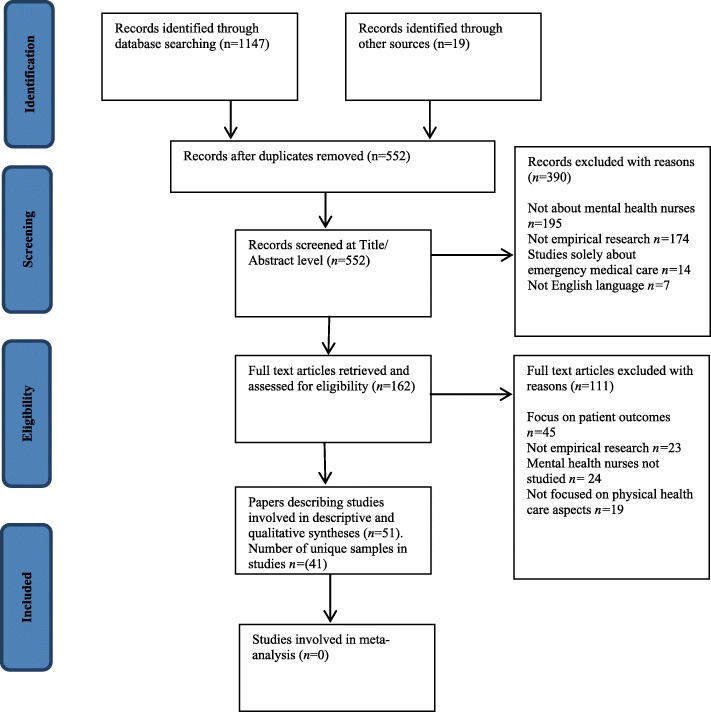
PRISMA study inclusion flowchart

The search strategy resulted in the inclusion of 41 study samples published in 51 papers (see Fig. 1) involving 7549 (*M*[*SD*] = 200.5[374.1], *Mdn =* 47, range 2 to 1899) mental health nurses and *n* = 213 mental health nursing students (*Mdn* = 33). Thirty-three samples included only nurses, of which 20 drew specifically on mental health nurses or nurses working in mental health settings only; eight samples were multidisciplinary. Four papers drew on two samples (i.e., two papers per study) while one sample featured in nine separate papers [30–38]. Studies were conducted in the UK (*k* = 17), Australia (*k* = 9), US (*k* = 4), Canada (*k* = 2), Qatar, Hong Kong, Japan, Jordan, Belgium, Norway, Israel, Turkey, India, and Taiwan (all *k* = 1); two studies were conducted internationally; first, in Qatar, Hong Kong, and Japan [19], and the US and Canada [39]. Studies were published between 1994 and 2018 (*Mdn* year of publication 2016, only *n* = 9 before 2010 and *n* = 1 before 2000).

### Study design

Eleven studies evaluated an intervention; of these, 10 utilised pre- post AB designs and one adopted a randomised controlled trial design. Other studies used cross-sectional survey or qualitative designs. Intervention studies sometimes incorporated additional qualitative or descriptive elements.

### Outcome measures

The most commonly used measure employed was the PHASe or some adaptation of it [11] in seven studies reported across eight papers [11, 19, 20, 40–44]. The PHASe comprises four factors: 1. Nurses’ attitudes to physical health care; 2. Nurses’ confidence to provide physical health care; 3. Nurses’ perceived barriers in providing physical health care; and 4. Nurses’ attitude towards smoking. Contact with study corresponding authors (Bressington, Chee, Haddad) resulted in acquisition of additional PHASe total and subscale information that was not included in the respective published study papers. Two other outcomes tools were used in two studies each, these being the purpose-designed survey measure of Howard and Gamble [45] subsequently used by Terry and Cutter [46], and Happell’s [33] own questionnaire adapted for use by Clancy et al. [40]. Most studies used purpose-designed tools. Many reported sufficient information to allow confidence about their internal reliability and face/content validity but there was little information about their measurement reliability, criterion validity, or sensitivity to change (see Additional file 5: Table S5). A small number of papers used existing validated measures [47–52] and these were generally the most robust tools (see Additional file 6: Table S6).

### Study quality

All *K* = 7 qualitative studies were rated very highly in terms of their quality on a 10-point assessment (*Mdn* = 9, range 9–10). Cross-sectional observational studies met a median of four of seven quality criteria (range two to six; mean[SD] 4.43[1.33]). Four of these provided an a priori sample size calculation and there was a lack of valid outcome measures in nine of the 21 studies. Overall risk of bias for cross-sectional studies was judged to be low for nine studies, unclear for six and high for six. The quality of interventional studies was generally the poorest (*Mdn* = 5, range 2 to 7 of 10 indicators). Only two were judged to be at low risk of bias (see Additional file 2: Tables S2, Additional file 3: Table S3, Additional file 4: Table S4, Additional file 5: Table S5 and Additional file 6: Table S6 for further details). Common omissions were, again, sample size justification, lack of repeat pre-baseline and follow up measures, and information about the representativeness of included samples.

### Study synthesis

#### Non-intervention studies

Studies examined physical healthcare in general (*k* = 24), sexual health (*k* = 4), smoking (*k* = 6), physical activity and healthy eating, nutrition - in particular the role of Omega-3 in diet, mild brain injury, and breastfeeding (all *k* = 1; see Table 1).

**Table 1:** Mental health nurses and physical healthcare (knowledge, experience, attitudes, education) Included studies

Study and [data collection year]	Location	Study design and focus	Data sources/ outcomes/ analysis	Sample	Intervention/ Exposure	Level of analysis	Main findings
*MHNs and physical healthcare: Cross-sectional and qualitative studies*							
Bressington et al. [19] [2016–17]	Qatar, Hong Kong, Japan	Cross-sectional survey. Physical healthcare.	Questionnaire: PHASe [11] and Japanese translation	*N =* 481 MHNs (39% response rate) 57% F; < 5-yrs in MH 14%.	Routine practice	National/ Inter-national	Nurses’ attitudes and confidence predict physical health management participation. Training needs perceived across registration and nationality; especially cardio-metabolic health.
Brimblecombe et al. [53] [2005]	England	Mixed. Cross-sectional, qualitative. Physical healthcare.	Purpose-designed tool. Content analysis. Researcher categorisation and inferential statistics.	*N =* 326 submissions from Higher Education(HE) and care organisations, open meetings, individual and MHN groups (*n =* 119)	Consultation document	National	Promoting healthy lifestyle most commonly mentioned by HE organisations. ‘Physical assessment skills’ were required according to open meetings and NHS organisation respondents but significantly less so by individual or groups of MHNs.
Ҫelik Ince et al. [56] [2017]	Turkey	Qualitative. Physical healthcare.	Semi-structured interviews on physical health care	*N* = 12 mental health nurses	Routine practice	Two hospitals	Themes: 1. Barriers to physical healthcare; 2. Current physical healthcare practices; 3. Motivators for providing physical healthcare; 4. Needs if physical health care is to improve.
Chee et al. [41] [2015]	Australia	Cross sectional survey. Physical healthcare in First Episode Psychosis care	Questionnaire: Amended PHASe [11]	*N* = 207 MHNs and Generalist nurses working in mental health services	Routine practice	National	Varying levels of physical health practice. See Table 2
Clancy et al. [40] [not reported]	Australia	Cross sectional survey. Physical healthcare.	Questionnaire: Adapted PHASe [11]; (Happell et al. [30]. Additional items.	*N =* 385 clinicians and managers (*n* = 198 nurses 51.4% on a 31% response discipline- rate)	Routine practice	Service	MHNs rated as having strong role legitimacy (monitoring, motivating, supporting) in relation to physical health interventions, medication effects, substance use, and sexual health both in absolute terms and relative to most other disciplines.
Delaney et al. [54] [not reported]	US	Cross-sectional survey. Physical healthcare.	Questionnaire. Researcher categorisation of responses and descriptive statistics.	*N =* 1899 Advanced Practice MHNs	Routine practice	National	Respondents rarely identify physical assessment (< 4.0%) or pathophysiology (0.5–5.0%) skills as a deficit.
Ganiah et al. [42] [not reported]	Jordan	Cross-sectional survey. Physical healthcare.	Questionnaire: PHASe [11]. Arabic translation.	*N =* 225 MHNs; 40.9% F; *M* experience 6.7-yrs	Routine practice	National	Significant but small correlations between participants’ attitudes and: reported physical healthcare practice (*r* = 0 .39); years in mental health care (*r* = − 0.207); *M n* assigned patients per nurse (*r* = − 0.18)^a^
Happell et al. [30] [2012]	Australia	Cross-sectional survey. Physical healthcare.	Questionnaire: Modified PHASe [11]	*N =* 643 see 5.	Routine practice	National	Varying levels of physical health practice and attitudes. See Table 3.
Happell et al. [31] [2012]	Australia	Cross-sectional survey. Physical healthcare.	Questionnaire: Strategies for Improving Physical Health of Consumers with Serious Mental Illness. Adapted PHASe [11]	*N =* 643 MHNs (22% response); 72.7% F; < 10-yrs in MH 15.7%	Routine practice	National	Training priorities: cardiovascular health (76.2%); diabetes (71.4%); assessment of physical illness (69.2%); weight management interventions (68.6%); exercise (66.4%); healthy eating (64.2%); smoking cessation (63.0%); reproductive health (62.4%); sensitive health issues (62.1%).
Happell et al. [32] [2012]	Australia	Cross-sectional survey. Physical healthcare.	Questionnaire: Rate strategies for improving patients’ physical health	*N =* 643 see 5.	Routine practice	National	High endorsement of nurse-based strategies (lifestyle programmes, screening), less for reducing antipsychotics. Most value attached to colocation of mental and physical health services, training GPs.
Happell et al. [72] [2012]	Australia	Qualitative. Physical healthcare.	Focus groups: What training needed to address physical health of patients?	*N =* 38 MHNs; MH experience < 1 to 22-yrs (*Mdn =* 11-yrs)	Routine practice	Region	Training priorities: physical health care: physical assessment, physical observations, diabetes. Strong beliefs about modes of training, access to training, and organizational commitment.
Happell et al. [73] [2012]	Australia	Qualitative. Physical healthcare.	Focus groups. Topics: Physical illness: physical health of patients; care responsibility; patient engagement	*N =* 38; MH experience < 1 to 22-yrs (*Mdn =* 11-yrs)	Routine practice	Region	Common experience of comorbid physical/mental illness in clients. Important for health-care services to treat and prevent physical illness. Divergent views on nurses’ capacity to contribute to better outcomes.
Study and data collection year	Location	Study design and focus	Data sources/ outcomes	Sample	Intervention/ Exposure	Level of analysis	Main findings
Happell et al. [33] [2012]	Australia	Cross-sectional survey. Physical healthcare.	Nurse Collaboration With Other Staff on the Physical Health of Consumers questionnaire	*N =* 643 see 5.	Routine practice	National	Physical health most frequently discussed with GPs, psychiatrists, case managers (*Mdn =* ‘Often’); least with OTs and SWs (*Mdn =* ‘Never’). Nurses who discuss physical health with one other profession are more likely to discuss it with a second type (true for 52/56 possible (range *r* = 0.21 to 0.59 ^a^).
Happell et al. [34] [2012]	Australia	Cross-sectional survey. Physical healthcare.	Adapted PHASe [11] plus new items.	*N =* 643 see 5.	Routine practice	National	Physical health care was explained by self-reported nurse views on patient health, rights and nurse role ideal (‘nurses should be involved in physical health care’), and organisational factors. The latter may be more important in determining physical health care
Happell et al. [35] [2012]	Australia	Cross-sectional survey. Physical healthcare.	Questionnaire domains: 1.Perceived Relative Health; 2. Healthcare Arrange-ments; 3. Value of Physical Healthcare Initiatives; 4. Cardio-metabolic Health Nurse (CHN) support	*N =* 643 see 5.	Routine practice	National	Predictors of CHN support: belief in GP physical healthcare neglect, interest in training; higher perceived value of improving physical health care (standardized β coefficients 0.11. 0.14, and 0.27 respectively)^b^
Howard & Gamble [45] [not reported]	UK	Cross-sectional survey. Physical healthcare.	Purpose-designed self-report questionnaire	*N =* 37 ward-based MHNs (47% response); Qualified < 5-yrs 43%	Routine practice.	Service	Gap between perceived responsibility and practice highlighting need for role clarification and skills training
Mwebe [55] [not reported]	UK	Qualitative. Physical healthcare.	Semi-structured interviews on physical health monitoring	*N =* 11 MHNs; < 10-yrs length of service 72.7%	Routine practice.	Service	Commitment to physical health screening and monitoring role. Themes: current practice; perceived barriers; educational needs; strategies to improve
Nash [71] [not reported]	UK	Cross sectional survey. Physical healthcare.	Purpose designed self-report questionnaire	*N =* 179 MHNs (53% response); *M-yrs* qual-ification 3.5, < 10-yrs 58%	Routine practice	Service	58% experienced in physical health care giving; 55% received training; 71% currently providing physical care: diabetes (53%), cardiac (23%), chest (19%), skin (32%), analgesia (32%), detox (13%). Training needs: 96% willing to attend skills training.
Osborne et al. [47] [not reported]	Australia	Cross-sectional survey. Physical assessment skills	Physical Assessment Skills Inventory [74, 75] Barriers to Registered Nurses’ Use of Physical Assessment Scale [76]	*N* = 433 registered nurses including 34 (7.8%) mental health nurses; 90.8% F; < 3-years experience as RN 10.8%.	Routine practice	Hospital	Mental health nurses use fewer (7/21) ‘core’ physical assessment skills (those used on average every day) than nurses in other specialties (surgical; maternity; medical; oncology; mean = 10.2). The skills most regularly used by mental health nurses (measuring temperature 73.5%, measuring SpO_2,_76.4%, measuring blood pressure 70.6%) are less commonly used than by all other nurses ((85.6, 85.4, and 75.4% respectively).
Phelan [77] [not reported]	UK	Audit. Physical healthcare.	Physical health care (PHC) check tool	60 community-based clients. PHC completed by MHNs (68.3%)	Routine practice	Team	More problems in this group of patients than in an audit of records from a similar team not using PHC. Tool seems to help nurses identify problems.
Robson & Haddad [11] [2006–7]	UK	Cross-sectional survey. Physical healthcare.	Questionnaire: PHASe	*N =* 585 MHNs; 62.2% F	Routine practice	Region	Varying levels of physical health practice and attitudes. See Tables 2 and 3.
Robson et al. [20] [2006–7]	UK	Cross-sectional survey. Physical healthcare.	Questionnaire: PHASe [11]	*N =* 585 MHNs see 10	Routine practice	Region	Varying levels of physical health practice and attitudes See Tables 2 and 3
Shuel et al. [78] [2007–8]	UK	Audit/ Survey Physical healthcare.	Serious Mental Health Improvement Profile (HIP), short semi-structured interviews	*N =* 31 patients seen by two HIP-trained MHNs	Use of HIP in routine practice	Service	The HIP used by MHNs identifies some physical issues. Authors recommend that training is required if they are to use it effectively.
Wynaden et al. [44] [2014]	Australia	Cross-sectional survey. Physical healthcare.	Questionnaire: PHASe	*N =* 170 nurses in public mental health services	Routine practice	Three services	Workplace culture influences the physical health care provided. Nurses are uncertain about where there priorities lie.
Study and data collection year	Location	Study design and focus	Data sources/ outcomes	Sample	Intervention/ Exposure	Level of analysis	Main findings
*MHNs and physical healthcare: Longitudinal/Intervention studies*							
Fernando et al. [66] [not reported]	UK	Longitudinal AB. Physical healthcare.	Purpose designed questionnaire	*N =* 63 nurses and junior doctors (15[24%] MHNs)	Physical/ mental health simulation	Region	Total knowledge, attitudes, and confidence scores improved but no data specific to delirium.
Haddad et al. [43] [not reported]	UK	Longitudinal AB. Physical healthcare.	Questionnaire: PHASe [11]	*N =* 49 (response 60%); < 10 years since qualification 60%. Low secure mental health unit.	Patient personal health plan Workshop.	Service	Modest (*d =* .09) statistically-significant improvement in staff knowledge scores and attitudes to involvement in physical health care. See Tables 2 and 3
Hemingway et al. [68] [not reported]	UK	Longitudinal AB. Physical healthcare.	Multiple choice format knowledge questionnaire	*N =* 204 (*n =* 89 registered and 115 students). *Mdn* age 39-yrs	5 × 1-d physical healthcare workshops	Region	All knowledge areas significantly improved from A to B. Effect sizes *d* = 1.4 wound care to 4.6 diabetes via 1.7 Oral health, 2.79 IM injections and 2.74 HIP). Almost all participants satisfied or very satisfied^c^
Terry & Cutter [46] [not reported]	UK	Longitudinal AB plus qualitative. Physical healthcare.	Purpose-designed self-report questionnaire [45]	15 MHNs in AB study, 5 in focus group; < 3-yrs in post 23.1%	Physical care degree module	Module cohort.	*M* confidence 97.9 T1 to 121.1 T2, *p* < .001 *r* = 0.98. Improvements on 25/39 questionnaire items. Focus groups: physical healthcare becoming more important in practice. Lack info and want more knowledge. ^a^
White et al. [67] [not reported]	UK	Longitudinal AB. Physical health.	Knowledge of/attitudes to (10 MCQs) physical health in severe mental illness	*N =* 38 matched pairs 78.3% F; < 5-yrs in health care 47.9%	2.5 h physical health work-shop. HIP	Region	Statistically significant knowledge-gain post-workshop (*d* = 1.16). Participants satisfied with content and willing to apply learning^c^
*MHNs and care for specific physical health issues: Cross-sectional and qualitative studies*							
Artzi-Medvdik et al. [48] [2006]	Israel	Cross-sectional survey. Breastfeeding in women with schizophrenia diagnosis.	Knowledge and attitudes to breastfeeding [79]. Adapted Attribution Questionnaire-27 [80]	*N =* 110 (response 57.9%) F RNs practicing in psychiatry/obstetrics (MHN *n =* 37; *M* yrs. registered 6.64]; Midwifery *n =* 40; postpartum care *n =* 33).	Routine practice	MHNs vs. Midwives vs. Post-partum care	Positive attitudes to breastfeeding in mothers with schizophrenia in 70% of respondents and to women with schizophrenia. MHNs significantly less knowledge re: breastfeeding, poorer attitudes to breastfeeding, more knowledge about schizophrenia. Predictors of positive attitude towards breastfeeding in women with schizophrenia: academic education (OR = 2.87), fear of schizophrenic patient (OR 0.27), extended schizophrenia-related knowledge (OR = 0.35)^d^
Dorsay & Forchuk [59] [not reported]	Canada	Cross-sectional survey. Sexual health	Purpose-designed survey questionnaire	*N =* 66 MHNs (response 20%)	Routine practice.	Service	Participants knowledgeable and competent. Most common sexual issues were abuse, contraception, STDs. Patient interviews suggested most had not been appropriately engaged in conversation.
Happell & Platania-Phung [35] [2012]	Australia	Cross-sectional survey. Cardio-vascular health promotion	Adapted PHASe [11] plus new items.	*N =* 643 see 5.	Routine practice	National	Perceived patient–nurse collaboration as a dual-determinant of nurse perceived barriers and self-reported health promotion to patients with SMI. Perceived barriers to consumer lifestyle change did not predict health promotion. The effects of nurse–patient collaboration were significant, but small.
Happell et al. [36] [2012]	Australia	Cross-sectional survey. Cardio-metabolic Health Nurse Role	133 open comments about the role of the CHN	*N =* 643 see 5.	Routine practice	National	Nurses see the specialist role as suitable and valuable for mental health services. Some concerns about role fragmentation with increasing specialty.
Happell et al. [38] [2012]	Australia	Cross-sectional survey. Dental health.	Adapted PHASe [11] plus new items.	*N =* 643 see 5.	Routine practice	National	The majority of nurses considered the oral and dental conditions of people with serious mental illness to be worse than the wider community. When compared with a range of significant physical health issues (e.g. cardiovascular disease)
Hughes & Gray [63] [not reported]	UK	Cross-sectional survey. HIV/AIDS	Purpose-designed questionnaire	283 Mental health workers (44% response). 51% nurses	Routine practice	Region	Sexual health promotion: part of role (80.3%); mandatory training required (78.3%); comfortable with LGBT issues (71.3%). People with SMI should be discouraged from having sex (1.8%); Discussing sexual activity encourages it (4.3%); ok to test HIV status without patient consent (4.6%).
Johannessen et al. [62] [not reported]	Norway	Qualitative. Omega-3/ Nutrition.	Questionnaires (students) and interviews	*n =* 50 student nurses; *n =* 20 tutor nurses; *n =* 5 psychiatrists.	Routine practice	Region	Nutrition considered important but few evaluations are made. Lack of Omega-3 knowledge. Unclear divisions of responsibility.
Klein & Graves [39] [2014]	US/ Canada	Cross-sectional survey. Mild brain injury (MBI).	Online survey questionnaire	*N =* 1049 nurse practitioners (23% response) inc. 139 MHNPs (84.3% F; < 5-yr as NP 25.4%)	Video of standardised MBI patient	National/ cross-border	MHN practitioners significantly less likely to: have had relevant training, think the injury is a concussion, use standardized instruments. Reported discomfort with the survey as due to knowledge deficit. Less likely to have had relevant training.
Study and data collection year	Location	Study design and focus	Data sources/ outcomes	Sample	Intervention/ Exposure	Level of analysis	Main findings
Magor-Blatch & Rugendyke [50] [not reported]	Australia	Cross-sectional survey. Smoking.	Attitudes toward Smoking Scale [81] Shore et al	*N =* 98 Mental Health Practitioners (*n =* 9 nurses) all settings	Routine practice.	Region	44.9% approved smoke-free policy. Attitudes to smoking restrictions (*r* = 0.35), concerns re: second hand smoke (*r* = 0.37), and to relationships with smokers (*r* = .39) associated with smoke-free agreement. Only attitudes pro- (positive relationship), and anti- the smoking ban (negative relationship) predicted ban support^a^
Nash [82] [not reported]	UK	Cross-sectional surveyDiabetes	16-item questionnaire	*N =* 138 MHNs (response 63%); qualified< 3-yrs 26%;	Routine practice	Service	69% currently providing diabetes care (most daily or weekly or bi-weekly 65%) Need for training in all aspects of diabetes care. 64% had not received training, 86% required further training.
Parel et al. [65] [Not stated]	India	Cross-sectional survey. Smoking.	Purpose-designed survey questionnaire.	*N* = 45 nurses in a psychiatric department.	Routine practice	Department	Moderate or greater knowledge about tobacco smoking and smoking cessation among participants. Cessation-training and attitudes to cessation negatively associated.
Quinn et al. [83] [not reported]	Australia	Qualitative. Sexual health	In-depth 1:1 interviews about experience of discussing sexuality with patients.	14 MHNs; 57% F; MHN experience 2–39 yrs. (*M* = 14.9)	Routine practice.	Service	Common reference to: sexual function assessment, psychotropic side-effects, patient embarrassment, and pros and cons of information. Sexual side effects recognised as impacting on medication adherence but most did not discuss it with patients.
Quinn et al. [60] [Not stated]	Uk & Australia	Cross-sectional survey. Sexual health care/	Purpose-designed survey questionnaire. Amended from Hughes and Gray [63]	*N* = 303 (*n =* 219 and 84 from Australia and UK respectively)	Routine practice	International	The results demonstrated that mental health nurses do not routinely include sexual health in their practice and are poorly prepared in knowing what to do with a sexual health issue, and what services to assist patients to use.
Sharma et al. [64] [not reported]	Australia	Cross-sectional survey. Smoking.	Online national survey questionnaire based on Ford et al. [84]	*N =* 267 mental health clinicians (22.8% nurses)	Routine practice	National	Compared with a reference category of medical practitioners, nurses were only significantly less likely to arrange follow up of smoking cessation interventions but not to ask, assess, advise, or assist. Training in smoking cessation associated with more cessation-related helping behaviour. Most believe harm reduction approaches to smoking cessation are effective.
Sharp et al. [58] [not reported]	US	Cross-sectional survey. Smoking.	Questions assessing intervention skills followed Ask–Advise–Assess–Assist–Arrange recommendations [85]	*N =* 1381 MHNs (approx. 33% response); < 5-yrs experience in MHN 17.2%	Routine practice	National	Most nurses assessed patients for smoking; fewer advised against smoking, referred for cessation, or delivered cessation interventions. More knowledgeable/self-efficacious nurses referred patients to smoking cessation resources (*d* = 0.41 to 0.8) or provided intensive interventions (*d* = 0.45 to 0.73); those with cessation- consistent beliefs more likely to refer (*d* = 0.48 to 0.49) or provide intervention (*d* = 0.49–0.90)^c^
Verhaege et al. [61] [not reported]	Belgium	Qualitative. Health promotion.	Focus groups (staff) interviews (patients)	*N =* 17 MHNs; *N =* 15 patients homeless service	Routine practice	Service	Benefits of physical and mental health identified, but barriers to integrating healthy lifestyles into patients’ lives: lack of time and personal views and attitudes towards health promotion were important.
*MHNs and care for specific physical health issues: Longitudinal/intervention studies*							
Happell et al. [36] [not reported]	Australia	Longitudinal AB survey. Cardio-metabolic health.	14-item questionnaire	*N =* 42 nurses initially and *N =* 21 at follow-up.	Introduction of a CHN	Service	Nurses initially supportive of the role. 6-month trial of a CHN reduced ambivalence. Only one of 14 items statistically significant A CHN would help prevent onset of cardio-metabolic disorders in patientss; greater proportion gave a *negative* response at post-intervention (*d* = 0.59)^c^
Hemingway et al. [70]	UK	Longitudinal AB. Diabetes	MCQ 12 items. Course evaluation questionnaire.	26 student nurses and 9 qualified staff.	See 36		*M* improvement *d* = 1.37. Both students and qualified improved equally. Course evaluated well. ^c^
Hemingway et al. [69] [not reported]	UK	Longitudinal AB plus qualitative element. Diabetes	Custom MCQ 13 items; 10-item evaluation questionnaire. Content analysis of open ended questions.	*N =* 48 (22 students, 26 qualified)	DVD, present-ations, skills sessions.	Region	*M* (SD) Pre- 5.9(2.17) Post 7.04(1.85), *p* < 0.01 (*d* = 0.56) Course evaluated highly. Themes: Satisfaction; Suggestions to improve; Use of a life story; Clinical perspective.
Study and data collection year	Location	Study design and focus	Data sources/ outcomes	Sample	Intervention	Level of analysis	Main findings
Hunter et al. [49]	UK	Mixed. Longitudinal AB. Qualitative. Obesity.	Nurses Attitudes towards Obesity and Obese Patients Scale [86]. Focus groups.	39/205 eligible participated pre-test and 29/39 completed both Pre- and post-)	Simulation ‘bariatric empathy suits’.	Student cohort	NATOOPS α acceptable overall. Factor 5 0.541/0.414 at pre−/post. Pre- post differences on F1 F2 and F5. No differences on between group attitudes. Qualitative themes: Physical impact of the suit; psychosocial impact of the suit; thinking differently; simulation as learning experience; challenges and recommendations.
Sung et al. [51] [not reported]	Taiwan	Stage 1: Qualitative. Stage 2: RCT. Sexual health.	1.Focus Group; 2. *Knowledge of sexual healthcare scale*; *Attitude toward sexual healthcare scale*. *Self-efficacy for sexual healthcare scale*:	Stage 1: 16 nurses, *M* clinical experience 15.9-yrs, 100% F. Stage 2: *N =* 117 59 Experimental 58 Control. *n* MHNs unclear: allocation stratified to ensure representation.	Stage 1: None. Stage 2: Sexual healthcare training 16-h over 4-weeks.	Service	Stage 1: themes: a) Views and experience in dealing with sexual healthcare b) Expectations re: training. Stage 2: Experimental group significant improvements in knowledge (*d =* 1.02), attitude (*d* = 0.67), and self-efficacy (*d =* 1.02). Relative to controls, they made significantly greater knowledge improvements (β = − 0.12, *p* < 0.01) and attitudes (β = − 0.25, *p* < 0.05), but not self-efficacy (β = − 0.33, *p* = 0.18). No psychiatric versus other ward-type effect^b,c^
Wynn [52] [not reported]	US	Longitudinal ABDiabetes.	Clinical judgment rubric [87]. Diabetes-related medical transfer.	*N =* 20 MHNs in veterans mental health hospital	Simulations re diabetes care.	Service	Statistically significant pre post improvement scores on clinical judgment (*d* = 4.8). Proportion of medical emergency reports involving diabetes fell from 55 to 20% in post-intervention month.

^a^Pearson’s *r* Small = 0.3, Moderate = 0.5, Large = 0.7; ^b^Standardised β coefficient outcome variable rises by stated amount for each 1 SD unit change in the predictor variable; ^c^*d* = Cohen’s *d* 0.2 Small 0.5 Medium 0.8 Large effect size ^d^OR Odds Ratio relative risk of the predictor variable with the reference variable e.g. extended knowledge associated with positive attitudes OR 0.35 means a person with extended knowledge is only 35% as likely to have positive attitudes than someone without extended knowledge

With regards to studies using the PHASe, of all possible comparisons across studies (see Tables 2 and 3), the mean score of the study sample differed significantly from the reference sample [11] on 13 out of 21 (61.9%) subscale and three of four total score combinations (75.0%). Analysis revealed poorer attitudes compared to the reference sample on all three of the significantly poorer attitude scores on 10/17 (58.9%) subscale comparisons, and better attitudes on three (14.3%). However, the reference group only outperformed the other studies on two of the eight possible comparisons on the subscales ‘Physical Healthcare’ and ‘Confidence in Providing Physical Healthcare’ and was poorer for three comparisons. The PHASe total score difference was greatest (large effect size) between the reference sample and Chee et al’s [41] Australian sample (Cohens *d* = 1.13) followed by Bressington et al’s [19] Japanese mental health nurse sub-sample (*d* = 0.72). For subscale scores, effect sizes for differences were also largest between the reference sample and that of Chee et al. [41]. Effect sizes were in favour of the reference sample on the attitudes to smoking and barriers to physical healthcare subscales (*d* = 1.48 and 1.78 respectively). Next largest were differences between Haddad et al’s [43] sample also on the barriers to healthcare (*d* = 0.93) and attitudes to smoking subscales (*d* = 1.01). On this occasion differences were in favour of Haddad et al’s [43] sample. Attitudes to smoking were more favourable than the reference sample in two studies, comparable in one and poorer in two.

**Table 2:** PHASe *M* (*SD*) across subscales and totals by study and comparisons with reference study [11]

	Physical health care *M* SD		Confidence to provide physical health care *M* SD		Nurses’ perceived barriers to delivering physical healthcare *M* SD		Nurses’ attitudes to smoking*M* SD		PHASe Total *M* SD	
Bressington et al. [19] All	34.39****˅	5.20	21.79* ˅	4.07	20.43**** ˅	4.06	19.07****˄	3.20	95.68****˅	11.81
Qatar	35.5^NS^	5.45	24.69**** ˄	2.71	19.71**** ˅	4.32	18.00** ˄	3.07	97.89 *˅	8.93
Hong Kong	34.03**** ˅	5.83	23.29** ˄	2.89	20.31**** ˅	4.37	19.38**** ˄	3.23	97.01 **˅	11.60
Japan	33.89**** ˅	4.37	18.71**** ˅	3.46	21.02**** ˅	3.54	19.58**** ˅	3.11	93.2****˅	8.29
Chee et al. [41] ^a^	36.87^NS^	6.00	23.73^****^ ˄	2.50	17.24 ^****^ ˅	3.00	12.29^****^ ˅	3.50	90.13^****^ ˅	6.44
Ganiah et al. [42]	26.19 ^b^	3.34	23.46**** ˄	2.89	24.66*^	3.08	15.02**** ˅	2.7	89.33**** ˅	5.55
Haddad et al. [43]	39.86*** ˄	5.71	21.77^NS^	4.26	20.14**** ˅	3.73	20.88**** ˄	2.69	102.61 ^NS^	10.75
Wynaden et al. [44]	–	–	–	–	–	–	17.82^NS^	2.71	–	–
Robson et al. [11] (Reference sample)	36.62	6.43	22.31	3.63	23.92	4.34	17.62	3.71	100	10.53

^a^Data from personal correspondence. ^b^Scale 1 Based on 8/10 items (not breast examination or contraceptive advice) and therefore cannot calculate difference from reference *M* for this scale or PHASe total. **** *p* < .0001 *** *p* < .001 **p < .01 * *p* < .05 (Differs from reference group *M* ˄ favourably ˅ unfavourably)

**Table 3:**
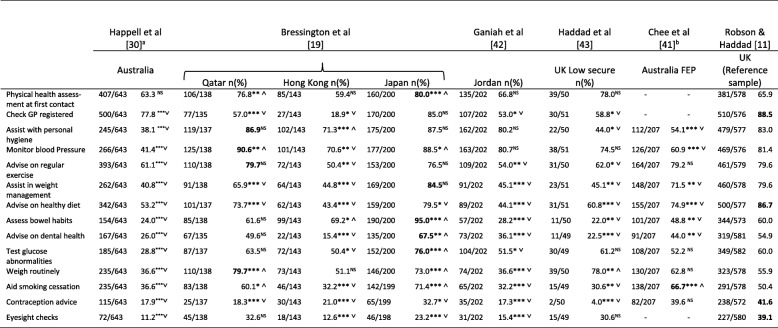
PHASe *n* and proportion who respond ‘Always’ or ‘Very often’ when asked with what frequency they conduct 14 physical healthcare-related items when working with mental health clients

*p < .05 **p < .01 ***p < .001 ˅ Compares unfavourably with reference sample; ˄ Compares favourably with reference sample; ^NS^ Not significant; FEP First Episode Psychosis. ^a “^How often do you undertake each of the following practices with consumers?” (response options: never, rarely, often, very often, always) vs. ‘My current practice involves… (response options: never, rarely, often, very often, always) ^b^No data presented for three items. Bold indicates the sample with the most favourable response by statement.

Regarding the level of self-reported involvement in aspects of physical healthcare the proportion of respondents in PHASe-studies answering ‘strongly agree’ or ‘agree’ to 14 items revealed considerable cross-sample differences. Of 95 possible comparisons between the reference study and others, 70 (73.7%) differed significantly. Of these, 86.7% compared unfavourably with the UK reference study, 13.3% favourably). The number of items per sample differing from the reference sample ranged from 7 to 13 (*Mdn =* 10). Japan [19] provided the only sample of mental health nurses whose responses compared favourably with the reference sample (7/10 significantly differing responses being more favourable in the Japanese sub-sample), while Ganiah et al’s [42] sample (0/11 favourable comparisons among significantly differing responses), Happell et al’s [30] (0/14 favourable comparisons), Chee et al’s [41] Australian sample (1/11 favourable comparisons), Haddad et al’s [43] UK sample (1/10 favourable comparisons) and Bressington et al’s [19] Hong Kong sample (2/12 favourable comparisons) all fared poorly. Items relating to checking GP-status, advising on exercise, weight management, healthy eating, contraception, and eyesight checks were all rated less favourably by at least two other samples (range 2 to 6, *Mdn* = 4) and more favourably by none compared with the reference sample. Only the item about ensuring patients have had their general physical health assessed on first contact with mental health services was rated more favourably by two samples and less favourably by none compared with the reference sample. For all other items there were item-level variations with no clear pattern.

The remaining non-intervention studies provide a mixed and sometimes contradictory picture. First, in terms of reported use of physical health care skills, Osborn et al’s [47] study revealed that nurses working in mental health settings in one large hospital were less likely to use physical healthcare skills than colleagues in medical, oncology, maternity and surgical settings. Further, they reported using a smaller range of relevant skills. In Howard and Gamble’s [45] survey, nurses’ responses indicated a gap between their perceived responsibilities for physical healthcare and their practice. Elsewhere, compared with those responding on behalf of healthcare and educational organisations, nurses were less likely to endorse their role in physical healthcare provision [53] and they reported very low levels of endorsement of related skills training need [54]. However, for others in more recent studies, they displayed a clear commitment to the physical healthcare role [55], and said they want more training [31, 56]. Further, nurses strongly endorsed their own role in physical health, sexual health, and substance abuse related care and were supported strongly by other healthcare professionals [40]. Across a series of linked surveys and qualitative studies, Happell et al. [30–37, 57] reported associations between nurses’ positive evaluation of the physical healthcare role and practicing aspects of it more commonly. In studies of nurses and specific physical healthcare-related activities there was a suggestion that respondents’ own values or beliefs might be more influential in determining their health-giving or advising behaviour in relation to smoking cessation [50, 58]. In relation to sexual health, both Dorsay and Forchuk [59] and Quinn et al. [60] have reported that nurses cite patient embarrassment as a reason for not asking patients about sexual side effects of antipsychotic medications. Lack of time, resources and knowledge were reported as barriers to providing advice and interventions regarding exercise and physical activity [61], Omega-3 [62]. Knowledge and attitudes to HIV/AIDS were generally good [63]. Finally, smoking-cessation training was associated with more smoking-cessation helping behaviour [64] though, counter-inuitively, training was negatively associated with attitudes to smoking cessation in a single study [65]. Further, Sharma et al’s [64] study compared the attitudes of mental health trained nurses and comprehensive/ generalist trained nurses working in mental health services: the most marked differences between the groups were on the smoking-related items with the former group expressing significantly more liberal views about smoking restrictions, more worrying attitudes about the benefits and utility of cigarette use as a therapeutic tool, and less confidence in the ability of mental health patients to quit smoking. This was particularly concerning in the study context which was about attitudes to physical healthcare with younger, first episode psychosis patients.

#### Intervention studies

Five studies focused on physical healthcare in general and six on specific issues (diabetes *n =* 3; sexual health, cardiometabolic health, obesity all *n =* 1). Ten evaluated an educational innovation, the exception being Happell et al. [35], who examined attitudes among nurses to the introduction of a specialist cardiometabolic health nurse role. Haddad et al. [43] examined the impact of the introduction of personal physical health care plans for patients on nurses’ physical healthcare attitudes alongside the delivery of a single educational session on physical healthcare assessment. The remaining nine studies evaluated educational interventions including three involving simulation and six involving didactic teaching, workshop-format or blended-learning approaches.

#### Simulation studies

Duration of interventions was 30 min [49] and1-day [66], while information was not provided by Wynn [52]. The mode of simulation delivery involved manikins [66], human actor as patient [66], software-based Human Person Simulator [52], and participant as ‘patient’ in which student participants wore a 15 kg bariatric empathy suit while undertaking everyday tasks in order to help them appreciate the experience of obesity [49]. Other simulations involved diabetes care [52], fractured leg in the context of a jump or fall in a patient with first episode psychosis, medical deterioration in the same patient following transfer to a psychiatric ward, and delirium [66]. Results indicated improved clinical judgement and reduced diabetes-related medical emergency reports [52], improved knowledge, attitudes, and confidence about physical healthcare [66], improved response to obese patients, characteristics of obese patients and supportive roles in caring for obese patients [49].

#### Non-simulation studie

Study duration ranged from a 2.5-h workshop on physical health [67] to a 20-credit bachelor’s degree level (equivalent to 200-h of taught and self-directed study and assessment completion) module on physical healthcare in mental health [46]. Non-simulation studies evaluated the introduction of personal health plans for patients in a low secure forensic unit together with a single educational session on physical health care for nursing staff [43]. Specific topics addressed included diabetes [68, 69], health assessment [46, 67], oral health, IM injectables [68], vital signs, blood readings, BMI measurement [46], and cardio-metabolic health [35, 57].

In Sung et al’s [51] RCT, nurses were allocated in a random stratified design to attend 8 × 2-h session about sexual healthcare over a period of 4-w or no intervention. Significant effects were detected in the experimental group relative to the control group for improvements in related knowledge and in attitudes, but not in self-efficacy. The study involved nurses employed both in medical and psychiatric wards (stratified allocation from both) and there was no reported effect of ward-type on outcomes. Pretest- posttest design intervention studies targeted at diabetes found greatly improved clinical judgment in relation to diabetes care and reduced diabetes-related emergency referrals [52] and similarly impressive improved diabetes-related knowledge [69, 70]. Improved attitudes to obesity, obese patients, and supportive roles in caring for obese individuals have been reported across a mixed group of participants and did not differ between mental health and other nurses [49]. and physical healthcare in general. Happell et al. [57] reported improved support for a specialist cardiometabolic nurse role following its introduction, however we find this conclusion is unwarranted since it is derived from statistical testing of 14-questionnaire items only one of which was found significant. Interventions aimed at physical healthcare in general found some impressive post- group improvements in knowledge [66–68], attitudes [66], and confidence [46, 66].

## Discussion

We have conducted a systematic review of the empirical literature about mental health nurses and their attitudes towards, knowledge about, and experiences of physical health care for patients. We took a broad approach to searching the literature and included interventional and observational studies involving real or simulated situations. We included studies involving mental health nursing students and multidisciplinary professional groups in addition to those including only mental health nurses. We contacted study authors to gain additional information and, for the studies using the PHASe [11] and this elicited significant, previously unpublished information. While we applied no time limits to our comprehensive search we found studies only from as early as 1994, only nine from before 2000, and the median year of publication was 2016. This means that there has been a welcome increase, which we described as a ‘mini-explosion’ in the Introduction, in related empirical work in recent years. The total number of nurses involved in studies, 7549, makes this to our knowledge one of the largest amalgamations of evidence gathered directly from mental health nurses.

However, the overall methodological quality of studies was somewhat limited, particularly interventional studies to improve mental health nurses’ physical healthcare assessment practices and skills. Nevertheless, while many of the included studies examine mental health nurses, and nurses working in mental health settings, this group comprises a heterogeneous collection of individuals of vastly differing experience, preparation, knowledge, and roles. As a result, it is not too surprising that some less well-researched areas have thrown up starkly different results. However, there is consistent evidence that there is a strong association between mental health nurses’ reported attitudes and their reported involvement in physical health care [19, 20, 42]. Similarly, that the nurses who value physical health care also report that they deliver more of it [30] and those who talk to at least one other discipline about their patients’ physical health do so with multiple professional groups [33]. Accordingly, fewer resources could be expended on answering these sorts of associational questions in the future.

Our conclusion is that it is now time for a new phase for mental health nursing research related to physical healthcare: efforts must be redoubled to focus on developing and testing interventions to improve nurses’ attitudes, knowledge, and skills. We must ensure that new studies are well-designed and rigorously conducted. More specifically, further research is required to build knowledge about whether the supposed benefits arising from this relationship translate into objectively better practice and indeed better patient outcomes. This would strengthen the case for training to improve attitudes and provide some urgency to better understand what interventions might deliver that outcome. Further, it appears that mental health nurses well-recognise that they require further skills and knowledge related to physical health care across a wide range of areas [19, 30, 31, 57, 71]. However, ambivalence and reluctance remains about embracing the change needed to achieve this [61].

The PHASe was used across multiple studies which allowed for some international and setting-specific comparison of nurses’ attitudes. We found that nurses’ self-perceived practices and attitudes differed significantly between samples from across the world. This, of course, may well reflect different approaches to mental health nurse preparation; for example, in Australia, all pre-registration nurses undergo the same core programme whereas in the UK mental health nursing is a specialist branch of pre-registration training. Therefore, results from Chee et al’s [41] recent study are enlightening since they reveal equivalent attitudes to physical healthcare specifically, more confidence in delivering physical healthcare but poorer scores in relation to barriers to physical healthcare delivery and smoking cessation. Given the non-equivalence of results on the attitudes to smoking subscale between Chee et al. [41] and Wynaden et al. [44], both conducted in Western Australia by related research teams, there are questions about the extent to which results are sample specific. Larger scale, representative data collection in Australia and New Zealand could therefore add significantly to the debate about nurses’ preparation for physical healthcare skills under different preparation regimes. As the PHASe authors’ note, the tool has not been subjected to tests of its stability or criterion validity and improvements in evidence for this would add significantly to the ability to draw sound conclusions from research using the tool. Findings from Osborne et al’s [47] large hospital-wide survey indicate that the gap in the physical health-related skills addressed by the PHASe is real and of concern.

Apart from the PHASe the literature is peppered with outcomes tools designed for single studies and with little evidence of anything other than face validity and internal consistency. Is it possible, we must ask, that this reflects that researchers are asking the wrong questions i.e., focusing overly on mental health nurses’ attitudes and self-proclaimed knowledge and efficacy when what is now required is a more robust approach to examining their actual knowledge and performance and, crucially, their impact on patient outcomes. Little seems to have been added to the literature on this since Hardy et al. [23] found no studies to include in their systematic review. Further, Haddad et al’s [43] study in a low secure forensic setting found nurses scoring favourably on PHASe subscales about attitudes to physical healthcare and to smoking compared with non-forensic nurses in the reference sample, suggesting perhaps that in a setting where length of stay is considerably longer then nurses have more opportunity to engage with patients in this aspect of care. Notably, however, nurses in the same sample compared unfavourably with the reference sample in terms of perceived involvement in actual physical healthcare, a somewhat contradictory finding.

For intervention studies, effect sizes were generally largest, and were in fact sometimes startlingly large, where interventions were targeted and outcomes were knowledge based (e.g., educational studies). This is unsurprising since educational interventions are generally evaluated against criteria that are specifically and directly addressed in the intervention. Outcomes tended to be measured immediately following the training [46, 52], but their long term retention is generally not known and neither is any practical beneficial change to practice. The apparent potency of these interventions requires further testing in randomized designs with appropriate follow-up periods.

Some study samples in the current review included non-nursing staff; though their occurrence and representativeness was too limited to allow robust conclusions to be drawn about the *relative* state of nurses’ knowledge and attitudes within the multidisciplinary team context. Given the current review explicitly focused on mental health nurses then further research exploring the multidisciplinary aspects of physical health care provision is warranted.

## Conclusion

Mental health nurses’ ability to provide routine physical healthcare has been highlighted in recent years. Recent literature provides a starting point for future research which must now concentrate on determining the effectiveness of nurse preparation for providing physical health care for people with mental disorder, determining the appropriate content for such preparation, and evaluating the effectiveness both in terms of nurse and patient- related outcomes. At the same time, developments are needed which are congruent with the needs and wants of patients. Perhaps what the included studies best demonstrate is that mental health nurses seem to realise that physical health care is part of their role.

## Additional files

Additional file 1: **Table S1.** Example PICO-style electronic literature search. Example literature search (DOCX 13 kb)

Additional file 2: **Table S2.** Controlled intervention evaluation study quality assessment. Study Quality Assessment (controlled intervention study) (DOCX 13 kb)

Additional file 3: **Table S3.** Cross-sectional, observational studies quality assessment (adapted from National Heart, Lung, and Blood Institute [26]. Study Quality Assessment (Cross-sectional and observational studies) (DOCX 16 kb)

Additional file 4: **Table S4.** Longitudinal uncontrolled intervention study quality assessment. Study Quality Assessment (uncontrolled intervention studies) (DOCX 14 kb)

Additional file 5: **Table S5.** Qualitative study quality assessment. Study Quality Assessment. (Qualitative studies) (DOCX 14 kb)

Additional file 6: **Table S6.** Outcome measure content and quality assessment. Quality assessment of outcomes measures used in studies. (DOCX 25 kb)

## Abbreviations

MeSH: Medical Subject Headings
PHASe: Physical Health Attitudes Scale for mental health nurses
PICO: Population Intervention Comparator Outcome
PRISMA: Preferred Reporting Items for Systematic Reviews and Meta Analyses
