# Supplementary material for: Mental health nurses’ attitudes, experience, and knowledge regarding routine physical healthcare: systematic, integrative review of studies involving 7,549 nurses working in mental health settings
Source: BMC Nurs. 2019 Apr 26;18:16. doi: 10.1186/s12912-019-0339-x (PMC6485121; doi:10.1186/s12912-019-0339-x)
Supplement: Supplementary file 1 — Table S1. Example PICO-style electronic literature search. Example literature search (DOCX 13 kb) [file 12912_2019_339_MOESM1_ESM.docx]

| **SUPPLEMENTARY MATERIAL Tables S1 to S6**  **N.B. All references in supplementary material refer to papers cited in the main manuscript with the exception of:**  †Mariani, B., Cantrell, Meakim, C. Prieto, P., & Dreifuerst, K.T. (2013). Structured debriefing and students' clinical judgment abilities in simulation. Clinical Simulation in Nursing, 9(5), e147-e145. doi: https://doi.org/10.1016/j.ecns.2011.11.009  ‡Adamson, K.A., Gubrud, P., Sideras, S., & Lasater, K. (2012). Assessing the reliability, validity, and use of the Lasater Clinical Judgment Rubric: Three approaches. Journal of Nursing Education, 51(2), 66-73. doi: https://doi.org/10.3928/01484834-20111130-03  **Supplementary Table S1: Example PICO-style electronic literature search** | | | |
| --- | --- | --- | --- |
| **Population** | **Intervention** | **Comparator** | **Outcome** |
| (Mental health OR Psychiatr*) AND Nurs* | ((Education OR Training OR Preparation OR Simulation OR Teaching OR Learning OR Educational Activities OR Training Programs OR Workshops OR Activities, Educational OR Training Activities OR Self Directed Learning OR Self Instruct* OR Self-Instruct*)  AND  (Physical health OR Physical monitoring OR Cardiovascular OR Heart Disease OR Heart Dis OR Cardiac Diseases OR Neurocirculatory Asthenia OR Blood Pressure Monitoring OR Blood Pressure Determination OR Heart Rate Determination OR Vital Signs OR Respiratory Function OR Respiratory Sounds OR Digestive System OR Liver Function Tests OR Pancreatic Function Tests OR Gastrointestinal Transit OR Blood Glucose OR Glucose Tolerance Test OR Hypoglycaemia OR Hyperglycaemia OR Diabetes OR Neurological OR Neurologic Examination OR Olfactometry OR Urological OR Kidney Function Tests OR Urinalysis OR Diagnostic Tests, Routine OR Traditional Pulse Diagnosis OR Medical History Taking OR Monitoring, Physiologic OR Drug Monitoring OR Haemodynamic Monitoring OR Monitoring, Ambulatory OR Neurophysiological Monitoring OR Telemetry OR Physical Examination OR Early Diagnosis OR Early Detection of Cancer OR Substance Abuse Detection OR Thermometry OR Obesity OR Malnutrition OR Body Weight OR Nutrition OR Reducing, Diet OR Nutritional Deficiency OR Undernutrition OR Heart Disease Prevention OR Eye Care OR Eye Disease OR Oral Health OR Mouth Diseases OR Sexual Health OR Breast Examination OR Mammogram OR Testicular Examination OR Prostate Examination OR Bowel Cancer test OR Cervical Smear OR Contraception OR Contraceptive Advice OR Family Planning OR Birth Control OR Exercise OR Acute Exercise OR Aerobic Exercise OR Exercise Training OR Exercise, Isometric OR Exercise, Physical OR Isometric Exercise OR Physical Activity OR Physical Fitness OR Physical Exertion OR Physical Activity OR Physical Fitness OR Sports OR Psychotropic Side Effects Physical OR Physical Health OR Health Promotion OR Promotion of Health OR Smoking OR Nicotine OR Tobacco OR Tobacco Use Disorder)) | None OR TAU Control* OR Treatment As Usual Control* OR Placebo Control* OR Wait* List Control* OR AB Design OR AB-Design OR Pre- Post Design OR Within Groups Design OR Within Subjects Design OR Within-Groups Design OR Within-Subjects Design | Attitude OR Belief OR Opinion OR Perception OR Experience OR Knowledge OR Skills OR Practice OR Practise OR Learning OR |
