# Supplementary material for: Mental health nurses’ attitudes, experience, and knowledge regarding routine physical healthcare: systematic, integrative review of studies involving 7,549 nurses working in mental health settings
Source: BMC Nurs. 2019 Apr 26;18:16. doi: 10.1186/s12912-019-0339-x (PMC6485121; doi:10.1186/s12912-019-0339-x)
Supplement: Supplementary file 2 — Table S2. Controlled intervention evaluation study quality assessment. Study Quality Assessment (controlled intervention study) (DOCX 13 kb) [file 12912_2019_339_MOESM2_ESM.docx]

**SUPPLEMENTARY MATERIAL Tables S1 to S6**

**N.B. All references in supplementary material refer to papers cited in the main manuscript with the exception of:**

†Mariani, B., Cantrell, Meakim, C. Prieto, P., & Dreifuerst, K.T. (2013). Structured debriefing and students' clinical judgment abilities in simulation. Clinical Simulation in Nursing, 9(5), e147-e145. doi: https://doi.org/10.1016/j.ecns.2011.11.009

‡Adamson, K.A., Gubrud, P., Sideras, S., & Lasater, K. (2012). Assessing the reliability, validity, and use of the Lasater Clinical Judgment Rubric: Three approaches. Journal of Nursing Education, 51(2), 66-73. doi: https://doi.org/10.3928/01484834-20111130-03

Supplementary Table S2: Controlled intervention evaluation study quality assessment

| Criteria | Sung et al [51] |
| --- | --- |
| 1. Was the study described as randomized, a randomized trial, a randomized clinical trial, or an RCT? | + |
| 2. Was the method of randomization adequate (i.e., use of randomly generated assignment)? | NR |
| 3. Was the treatment allocation concealed (so that assignments could not be predicted)? | NR |
| 4. Were study participants and providers blinded to treatment group assignment? | - |
| 5. Were the people assessing the outcomes blinded to the participants' group assignments? | NR |
| 6. Were the groups similar at baseline on important characteristics that could affect outcomes (e.g., demographics, risk factors, co-morbid conditions)? | - |
| 7. Was the overall drop-out rate from the study at endpoint 20% or lower of the number allocated to treatment? | + |
| 8. Was the differential drop-out rate (between treatment groups) at endpoint 15 percentage points or lower? | + |
| 9. Was there high adherence to the intervention protocols for each treatment group? | NR |
| 10. Were other interventions avoided or similar in the groups (e.g., similar background treatments)? | NR |
| 11. Were outcomes assessed using valid and reliable measures, implemented consistently across all study participants? | + |
| 12. Did the authors report that the sample size was sufficiently large to be able to detect a difference in the main outcome between groups with at least 80% power? | + |
| 13. Were outcomes reported or subgroups analyzed prespecified (i.e., identified before analyses were conducted)? | + |
| 14. Were all randomized participants analyzed in the group to which they were originally assigned, i.e., did they use an intention-to-treat analysis? | NR |
| Overall risk of bias: | High |
| Total: | 6/12 |

Key: + Condition achieved; NR = Not Reported
