# Supplementary material for: Mental health nurses’ attitudes, experience, and knowledge regarding routine physical healthcare: systematic, integrative review of studies involving 7,549 nurses working in mental health settings
Source: BMC Nurs. 2019 Apr 26;18:16. doi: 10.1186/s12912-019-0339-x (PMC6485121; doi:10.1186/s12912-019-0339-x)
Supplement: Supplementary file 3 — Table S3. Cross-sectional, observational studies quality assessment (adapted from National Heart, Lung, and Blood Institute [26]. Study Quality Assessment (Cross-sectional and observational studies) (DOCX 16 kb) [file 12912_2019_339_MOESM3_ESM.docx]

**SUPPLEMENTARY MATERIAL Tables S1 to S6**

N.B. All references in supplementary material refer to papers cited in the main manuscript with the exception of:

†Mariani, B., Cantrell, Meakim, C. Prieto, P., & Dreifuerst, K.T. (2013). Structured debriefing and students' clinical judgment abilities in simulation. Clinical Simulation in Nursing, 9(5), e147-e145. doi: https://doi.org/10.1016/j.ecns.2011.11.009

‡Adamson, K.A., Gubrud, P., Sideras, S., & Lasater, K. (2012). Assessing the reliability, validity, and use of the Lasater Clinical Judgment Rubric: Three approaches. Journal of Nursing Education, 51(2), 66-73. doi: https://doi.org/10.3928/01484834-20111130-03

| Supplementary Table S3: Cross-sectional, observational studies quality assessment (adapted from National Heart, Lung, and Blood Institute [26] | | | | | | | | | | | | | | | | | | | | | | |
| --- | --- | --- | --- | --- | --- | --- | --- | --- | --- | --- | --- | --- | --- | --- | --- | --- | --- | --- | --- | --- | --- | --- |
|  | Bressington et al [19] | Osborne et al [47] | Delaney et al [54] | Ganiah et al [42] | Nash [71] | Magor-Blatch & Rugendyke [50] | Sharp et al [58] | Nash [82] | Artzi-Medvik [48] | Dorsay & Forchuk [59] | Wynaden et al [44] | Happell et al [30-35,37] | Brimblecombe et al [53] | Robson & Haddad [11] Robson et al [20] | Hughes & Gray [63] | Klein & Graves [39] | Howard & Gamble [45] | Phelan et al [74] | Parel et al [65] | Chee et al [41] | Clancy et al [40] | Sharma et al [64] |
| Research question or objective in this paper clearly stated | + | + | + | + | + | + | - | - | + | + | + | + | + | + | + | + | + | + | + | + | + | + |
| Study population clearly specified and defined | + | + | + | + | + | - | + | + | + | - | + | + | + | + | + | + | + | + | - | + | + | + |
| Participation rate of eligible persons at least 50% | -* | - | - | + | + | NR | - | + | + | - | + | - | CD | + | - | - | - | NR | NR | + | - | + |
| Subjects selected or recruited from the same or similar populations and in the same time period | + | + | + | + | + | NR | + | + | + | + | + | + | + | + | + | + | + | - | - | + | + | CD |
| Sample size justification, power description, or variance and effect estimates provided | + | + | - | - | - | + | - | - | - | - | - | - | - | - | - | - | - | - | - | + | + | - |
| Inclusion/ exclusion criteria for participation pre-specified and applied uniformly to all | + | + | + | + | + | NR | + | + | + | + | + | + | + | + | + | + | + | - | - | + | + | + |
| Outcome measures clearly defined, valid, reliable, and implemented consistently | + | + | - | + | - | + | NR | - | + | - | + | + | NA | + | + | + | - | - | + | + | CD | CD |
| Overall risk of bias | L | L | U | L | U | H | H | H | L | H | L | L | U | L | L | L | U | H | H | L | U | U |
| Total (max 7) | 6 | 6 | 4 | 6 | 5 | 3 | 3 | 4 | 6 | 3 | 6 | 5 | 4 | 6 | 5 | 5 | 4 | 2 | 2 | 7 | 5 | 4 |

Key: + Condition achieved; - condition not achieved; NR = Not Reported; *>50% response rate for 2/3 sample subgroups. L = Overall low risk of study bias; H = Overall high risk of study bias; U – Unclear risk of study bias
