# Supplementary material for: Mental health nurses’ attitudes, experience, and knowledge regarding routine physical healthcare: systematic, integrative review of studies involving 7,549 nurses working in mental health settings
Source: BMC Nurs. 2019 Apr 26;18:16. doi: 10.1186/s12912-019-0339-x (PMC6485121; doi:10.1186/s12912-019-0339-x)
Supplement: Supplementary file 4 — Table S4. Longitudinal uncontrolled intervention study quality assessment. Study Quality Assessment (uncontrolled intervention studies) (DOCX 14 kb) [file 12912_2019_339_MOESM4_ESM.docx]

**SUPPLEMENTARY MATERIAL Tables S1 to S6**

N.B. All references in supplementary material refer to papers cited in the main manuscript with the exception of:

†Mariani, B., Cantrell, Meakim, C. Prieto, P., & Dreifuerst, K.T. (2013). Structured debriefing and students' clinical judgment abilities in simulation. Clinical Simulation in Nursing, 9(5), e147-e145. doi: https://doi.org/10.1016/j.ecns.2011.11.009

‡Adamson, K.A., Gubrud, P., Sideras, S., & Lasater, K. (2012). Assessing the reliability, validity, and use of the Lasater Clinical Judgment Rubric: Three approaches. Journal of Nursing Education, 51(2), 66-73. doi: https://doi.org/10.3928/01484834-20111130-03

Supplementary Table S4: Longitudinal uncontrolled intervention study quality assessment

|  | White et al [67] | Haddad et al [43] | Fernando et al [66] | Hemingway et al [68] | Wynn [52] | Happell et al [36] | Hemingway et al [69] | Hemingway et al [70] | Hunter et al [49] |
| --- | --- | --- | --- | --- | --- | --- | --- | --- | --- |
| Research question or objective in this paper clearly stated | + | + | + | + | + | + | + | - | + |
| Study population clearly specified and defined | + | + | - | + | - | + | + | - | + |
| Study participants representative of population | CD | + | + | CD | - | CD | CD | CD | + |
| Participation rate of eligible persons at least 50% | - | CD | CD | CD | CD | NR | NR | NR | CD |
| Sample size justification, power description provided | - | CD | - | - | - | - | CD | CD | CD |
| Intervention clearly described and delivered | + | + | + | + | + | + | + | + | + |
| Outcome measures clearly defined, valid, reliable, and implemented consistently | - | + | - | + | + | + | NR | NR | + |
| Blinding of assessors | - | NA | NA | NA | NA | NA | NA | NA | NA |
| Loss to follow up <20% | - | + | NR | + | + | - | - | NR | + |
| Statistical analysis of change pre- and post- intervention | + | + | + | + | + | + | + | + | + |
| Multiple pre-baseline and follow-up outcome measures | - | - | - | - | - | - | - | - | - |
| Group level interventions analysis accounts for individual level data | NA | NA | NA | NA | NA | NA | NA | NA | NA |
| Overall risk of bias | H | L | H | U | U | U | H | H | L |
| Total (max 7) | 4/11 | 7/10 | 4/10 | 6/10 | 5/10 | 5/10 | 4/10 | 2/10 | 7/10 |

Key: + Condition achieved; - condition not achieved; NR = Not Reported; L = Overall low risk of study bias; H = Overall high risk of study bias;

U – Unclear risk of study bias
