# Supplementary material for: Mental health nurses’ attitudes, experience, and knowledge regarding routine physical healthcare: systematic, integrative review of studies involving 7,549 nurses working in mental health settings
Source: BMC Nurs. 2019 Apr 26;18:16. doi: 10.1186/s12912-019-0339-x (PMC6485121; doi:10.1186/s12912-019-0339-x)
Supplement: Supplementary file 5 — Table S5. Qualitative study quality assessment. Study Quality Assessment. (Qualitative studies) (DOCX 14 kb) [file 12912_2019_339_MOESM5_ESM.docx]

**SUPPLEMENTARY MATERIAL Tables S1 to S6**

N.B. All references in supplementary material refer to papers cited in the main manuscript with the exception of:

†Mariani, B., Cantrell, Meakim, C. Prieto, P., & Dreifuerst, K.T. (2013). Structured debriefing and students' clinical judgment abilities in simulation. Clinical Simulation in Nursing, 9(5), e147-e145. doi: https://doi.org/10.1016/j.ecns.2011.11.009

‡Adamson, K.A., Gubrud, P., Sideras, S., & Lasater, K. (2012). Assessing the reliability, validity, and use of the Lasater Clinical Judgment Rubric: Three approaches. Journal of Nursing Education, 51(2), 66-73. doi: https://doi.org/10.3928/01484834-20111130-03

Supplementary Table S5: Qualitative study quality assessment

|  | Happell et al [72,73] | Mwebe [55] | Shuel [75] | Verhaege [61] | Johannesen et al [62] | Quinn et al. [83] | Celik Ince [56] |
| --- | --- | --- | --- | --- | --- | --- | --- |
| Clear statement of research aims | + | + | - | + | + | + | + |
| Qualitative method appropriate | + | + | + | + | + | + | + |
| Design appropriate to address research aims | + | + | + | + | + | + | + |
| Recruitment strategy appropriate to study aims | + | + | + | + | + | + | + |
| Data collected in a way that addressed the research issue | + | + | + | + | + | + | + |
| Researcher-participant relationship adequately considered | + | NR | + | + | + | + | + |
| Ethical issues considered | + | + | + | + | + | + | + |
| Data analysis rigorous | + | + | + | + | + | + | + |
| Clear statement of findings | + | + | + | + | + | + | + |
| Is the research valuable? | + | + | + | + | + | + | + |
| Total: (max 10) | 10 | 9 | 9 | 10 | 10 | 10 | 10 |

Key: + Condition achieved; NR = Not Reported;
