# Supplementary material for: Mental health nurses’ attitudes, experience, and knowledge regarding routine physical healthcare: systematic, integrative review of studies involving 7,549 nurses working in mental health settings
Source: BMC Nurs. 2019 Apr 26;18:16. doi: 10.1186/s12912-019-0339-x (PMC6485121; doi:10.1186/s12912-019-0339-x)
Supplement: Supplementary file 6 — Table S6. Outcome measure content and quality assessment. Quality assessment of outcomes measures used in studies. (DOCX 25 kb) [file 12912_2019_339_MOESM6_ESM.docx]

**SUPPLEMENTARY MATERIAL Tables S1 to S6**

N.B. All references in supplementary material refer to papers cited in the main manuscript with the exception of:

†Mariani, B., Cantrell, Meakim, C. Prieto, P., & Dreifuerst, K.T. (2013). Structured debriefing and students' clinical judgment abilities in simulation. Clinical Simulation in Nursing, 9(5), e147-e145. doi: https://doi.org/10.1016/j.ecns.2011.11.009

‡Adamson, K.A., Gubrud, P., Sideras, S., & Lasater, K. (2012). Assessing the reliability, validity, and use of the Lasater Clinical Judgment Rubric: Three approaches. Journal of Nursing Education, 51(2), 66-73. doi: https://doi.org/10.3928/01484834-20111130-03

| Supplementary Table S6: Outcome measure content and quality assessment | | | | | | | |
| --- | --- | --- | --- | --- | --- | --- | --- |
| **Study** | **Tool** | **Content** | **Internal consistency** | **Reliability** | **Content validity** | **Criterion validity** | **Responsiv-eness** |
| Artzi-Medvdik et al [48] | Knowledge and attitudes to breastfeeding [79] | personal and professional experience, knowledge, attitudes to schizophrenia (open-ended question subject to thematic analysis). Overall reliability α=0.62 for pilot 0.52 for study sample | NR | - | + | - | - |
|  | Adapted Attribution Quest-ionnaire-27 Corrigan [80] | Professional guidance of the women with schizophrenia about breastfeeding; and personal/ professional experience, know-ledge, attitudes towards breast-feeding among women *with* schizophrenia. Hebrew translation | NR | - | + | - | - |
| Bressington et al [19] | PHASe [11] | See Robson & Haddad [11]. Japanese translation for part of sample. Subscale 1) α=0.778 2) 0.889 3) 0.578  4) 0 .618 | + | - | + | - | - |
| Brimblecombe et al [53] | Purpose-designed questionnaire | Purpose-designed questionnaire by a multi-disciplinary reference group. 24 questions across four domains including ‘Physical’ health. Free text response. | - | - | + | - |  |
| Chee et al [41] | PHASe [11] | See Robson & Haddad [11]. Amended for use with people working with patients with First Episode Psychosis. Overall Cronbach’s α value 0.72, 0.83 for attitudes of nurses involved in physical health care, 0.74 for confidence of nurses in delivering physical health care, 0.69 for perceived barriers in delivering physical health care, and 0.60 for attitudes of nurses relating to smoking. | + | - | + | - | - |
| Clancy et al [40] | Strategies for Improving Physical Health of Consumers with Serious Mental Illness: (Happell et al [30] | See Happell et al [30] | - | - | + | - | - |
|  | PHASe (Robson & Haddad [11]) | See Robson & Haddad [11] | + | - | + | - | - |
| Delaney et al [54] | Purpose-designed questionnaire | “Did your graduate programme adequately prepare you for your role? How did it *not* prepare you?” | - | - | + | - | - |
| Dorsay & Forchuk [59] | Survey questionnaire | Staff perceptions of their comfort level, clinical competence, and frequency of involvement in dealing with sexual issues with patients. Audit of records to determine if sexual needs identified and care planned. Patient interviews. | - | - | + | - | - |
| Wynn [52] | Lasater 2007 clinical judgment rubric. | IC α=0.80 to 0.97 (Mariani et al 2013)†; IRR 0.889 to 0.96 (Adamson et al 2012)‡ | +. | + | + | - | - |
| Fernando et al [66] | Purpose-designed Self Evaluation and Course Evaluation Forms | Self-evaluation form. 21-item questionnaire examining knowledge (7 True/False items), attitudes (7 Yes/No items), and confidence (7 5-point Likert scales). Course evaluation form yes/no responses. Tools guided by literature and piloted prior to use. 12-item Course Evaluation Form Yes/No items plus open questions- | - | - | + | - | - |
| Ganiah et al [42] | PHASe (Robson & Haddad [11]) | See Robson & Haddad [11]). Arabic translation 1) α=0.626 (attitudes), 2) 0.912 (current practice), 3) 0.874 (training needs) | + | - | + | - | - |
| Haddad et al [43] | PHASe [11] | See Robson & Haddad [11] | + | - | + | - | - |
| **Study** | **Tool** | **Content** | **Internal consistency** | **Reliability** | **Content validity** | **Criterion validity** | **Responsiv-eness** |
| Happell et al [30] | Strategies for Improving Physical Health of Consumers with Serious Mental Illness: | List of physical health care initiatives provided and participants asked to rate “their potential for contributing to improving the physical health of consumers”. “Workplace training of nurses on physical care tasks and responsibilities” was one of the eight strategies included. Response option: Negative value/Counter-productive, No value, Little value, Moderate value, Significant value. | - | - | + | - | - |
| Happell et al [33] | Nurse Collaboration With Other Staff on the Physical Health of Consumers. | 1. “How regularly do you discuss physical health matters of consumers with: GP, Psychiatrist, Nurse Practitioner etc.” 2. Adapted PHASe (see Robson et al 2012) asking nurses to rate their level of physical health care for a range of 13actions: “How often do you undertake each of the following practices with consumers?”. Response for both 1 & 2: never, rarely, often, very often, always (1-5). | - | - | + | - | - |
| Happell et al [35] | 1.Perceived Relative Health; 2. Views on Healthcare Arrange-ments; 3. Value of Physical Healthcare Initiatives; 4. Support for a CHN role | 1.Perceived Relative Health: “How would you rate the physical health of consumers of MH services, compared to members of the wider community.” For: cardiovascular disease, diabetes, obesity, respiratory conditions, oral/ dental conditions (1 much worse to 5 much better). Cronbach’s α=.84. 2. Views on Healthcare Arrangements. Two statements (“GPs neglect physical health” and “MH system obligation”) respectively. about physical health in a mental health setting (1 strongly disagree to 5 strongly agree). 3. Value of Physical Healthcare Initiatives: “For strategy, please rate their potential for contributing to improving the physical health of consumers.” e.g., “general health bus,” “smoking cessation program,” and “mental illness stigma reduction program for primary care staff.” (1 “Negative value/ counter-productive” to 5“Significant value” 10 items). 4. Support for a Cardio-metabolic Health Nurse (CHN) role (14 items response: 1 strongly disagree to 5 strongly agree) | + | - | + | - | - |
| Happell et al [35] | 14-item questionnaire | Questionnaire to determine views of proposed specialist CHN role (Happell et al [31]). Responses on a 5-point Likert scale. Perceived relative health α=0.84 Views on healthcare arrangements (2 items only no α reported) Value of physical healthcare initiatives α= 0.74 Support of CHN α=0.87 | + | - | - | - | - |
| Hemingway et al [69] | Multi-choice test | MCQ 13-items testing knowledge of diabetes epidemiology, symptomatology, risk factors, prognosis, management. Plus 10-item workshop evaluation questionnaire. Qualitative content analysis of open ended questions. | - | - | - | - | - |
| Hemingway et al [68] | Purpose designed questionnaire | Multiple choice questionnaire regarding knowledge and confidence for physical health interventions. Course evaluation: Likert-style (scored from 1, least, to 3, most) items and spaces for open-ended comments. | - | - | - | - | - |
| Howard & Gamble [45] | Purpose-designed self-report questionnaire | Three sections: 1. participants’ views about their role and role of others (health care assistant, ward doctor, GP, Community care coordinator, other) in undertaking 41 physical health assessment and care management activities; 2. Participants’ level of confidence in undertaking physical health assessment and care management activities (*n*=41 items) on a 4-point scale (very confident to not at all confident(; 3. 12 questions about education and training. | - | - | + | - | - |
| Hughes & Gray [63] | Purpose-designed questionnaire | 42 statements, 5 subscales: Attitudes to sexual health promotion α=0.23 (12); Knowledge about HIV/AIDS α=0.43 (5); Knowledge about risk behaviours (α=0.62) and risk factors (α=0.66) for HIV infection (13); Glove wearing (α=0.75) (5); Reported sexual health promotion practice (7). Two open-ended questions about training and services needed. | - | - | + | - | - |
| Hunter et al [49] | Nurses Attitudes towards Obesity and Obese Patients Scale (Watson et al [86]). | Nurses Attitudes towards Obesity and Obese Patients Scale (Watson et al 2008). 5 subscales: response to obese patients, characteristics of obese individuals; controllable factors contributing to obesity; stereotypical characteristics of obese patients, and supportive roles in caring for obese patients. (VAS 0-100) α=0.857 | + | - | + | - | - |
| **Study** | **Tool** | **Content** | **Internal consistency** | **Reliability** | **Content validity** | **Criterion validity** | **Responsiv-eness** |
| Klein & Graves [39] | Online survey questionnaire | Including a standardized patient video with M/F (C1) adolescent actors demonstrating mild TBI symptoms from a sports-related (soccer) or leisure (hiking) injury (C2). Video symptoms matched to a standardized diagnostic tool. 25-item post-video survey examining: concussion, differential diagnosis, use of standardized concussion tools, recommendations for return to school the same day or reduced workload. | + | - | - | - | - |
| Magor-Blatch & Rugendyke [50] | Attitudes toward Smoking Scale (Shore et al [81]: | 1. Interpersonal relationships with smokers (α=0.87) 2. Laws and societal restrictions of smoking in public places (0.87) 3. health concerns (0.61). | + | - | + | - | - |
| Nash [82] | 16-item questionnaire | Whether they had received any specific training or experience in diabetes care; where and when any specific training had taken place; the impact of diabetes care on their current workload; the types of diabetes care given; type of training required. | - | - | - | - | - |
| Nash [71] | Purpose designed questionnaire | 16-item self-report questionnaire: items on past experience and training in physical health care, areas of current physical care-giving, impact of physical health care on workload, desired skills, and preferred training methods | - | - | + | - | - |
| Osborn et al [47] | Physical Assessment Skills Inventory (Giddens [77]; Birks et al [76]  Barriers to Registered Nurses’ Use of Assessment Skills (Douglas et al., [78]) | 133-item scale modified from previous versions. designed to explore registered nurses knowledge and frequency of use of physical assessment skills, grouped according to 15 body regions/systems. Participants are requested to indicate the frequency with which they performed each skill using a 6-point Likert response scale: 0 = I do not know how to do this skill, 1 = I know how to do this skill, but have never done this in my clinical practice, 2 = I perform this skill rarely (a few times during my career), 3 = I perform this skill occasionally (a few times a year), 4 = I perform this skill frequently in my clinical practice (every 2–5 times I work), 5 = I perform this skill regularly in my clinical practice (every time I work).  38-item, 7-factor scale modified for index study from previous work. Participants required to indicate the extent to which they agreed each item applied to their practice on a 5-point Likert response scale, ranging from 1 (strongly disagree) to 5 (strongly agree). Subscales: (1) reliance on others and technology, (2) lack of time and interruptions, (3) ward culture, (4) lack of confidence, (5) lack of nursing role models, (6) lack of influence on patient care, and (7) specialty area. | -  + | -  - | +  - | -  - | - |
| Parel et al [65] | Attitude Scale (Parel et al [65]) | Part-1 was designed to elicit the socio-demographic details of the subjects and some selected variables specific for the study (α=0.88). The part-2 consists of items to explore the knowledge level of nurse regarding prevention of tobacco use (α=0.86). | + | - | + | - | - |
| Phelan [74] | Physical Health Check | The PHC consists of 27 specific items, divided into 4 sections. 1. current medication and known illness, and lifestyle such as diet, exercise and smoking habits; 2. current physical symptoms; 3. physical investigations and health screening; 4. action plan where the practitioner and patient record the agreed actions arising from the assessment. | - | - | - | - | - |
| Robson & Haddad [11] | PHASe [11] | Four scales (1. Nurses’ attitudes to physical health care; 2. Nurses’ confidence to provide physical health care; 3. Nurses’ perceived barriers in providing physical health care; 4. Nurses’ attitude towards smoking), 28-items. Plus 14 questions on current physical healthcare related practice. IC: Whole scale α=0.765; Subscales: 1) 0.860, 2) 0.742, 3) 0.67, 4) 0 .61 | + | - | + | - | - |
| **Study** | **Tool** | **Content** | **Internal consistency** | **Reliability** | **Content validity** | **Criterion validity** | **Responsiv-eness** |
| Robson et al [20] | PHASe [11] | See Robson & Haddad [11] | + | - | + | - | - |
|  | Knowledge inventory | 12-item multiple choice knowledge inventory measure of staff knowledge devised by clinical and academic nursing staff and piloted with nurse academics | - | - | + | - | - |
| White [67] | Purpose designed questionnaire | Knowledge (10 MCQs) of and attitudes to (Likert scale) physical health in severe mental illness questionnaire | - | - | + | - | - |
| Sharma et al [64] | Modelled on Ford et al [84] | 1.Demographics; 2. And 3.practices related to smoking cessation; 4. Beliefs about tobacco harm reduction | - | - | + | - | - |
| Sharp et al [58] | Based on Ask–Advise–Assess–Assist–Arrange recommendations [85] | Questions assessing intervention skills followed Ask–Advise–Assess–Assist–Arrange recommendations (Morris et al 2009) for clinicians and agencies without ready referral access to tobacco cessation services. Plus whether they delivered more intensive tobacco dependence interventions (4 to 8 sessions exceeding 30 minutes of total contact time with pharmacotherapy and behavioural counselling). | - | - | + | - | - |
| Shuel et al [75] | Health Improvement Profile (HIP). | HIP – N/A Semi-structured interviews. Categories devised by researchers and qualitative data coded into them. | - | - | - | - | - |
| Sung et al [51] | Knowledge of sexual healthcare scale | 31 items, scored 0/1); IC α=0.81, IRR ICC=0.73 | + | + | + | - | - |
|  | Attitude toward sexual healthcare scale | 18 items: 6 comfort discussing sexual issues, 6 perspectives on professional role, 6 willingness to provide sexual information. Rated 0-5) α =0.92 | + | + | + | - | - |
|  | Self-efficacy for sexual healthcare scale | 22 items: 13 confidence discussing sexual matters, 5 confidence providing info, 4 self-adjustment of uncomfortable feelings (scored 1-5). α *=*0.97 | + | + | + | - | - |
| Terry & Cutter [46] | See Howard & Gamble [45] | Perceptions of confidence in undertaking physical health assessments and care (Howard & Gamble 2011). Measuring vital signs (α=0.85), checking blood results (1.0); recording BMI (0.75); assessment (0.9); provision of advice (0.92 minus 1 item) 1 x Focus group. | + | - | + | - | - |

Key: + Condition achieved; - condition not achieved; NR = Not Reported
